# Does PCNA diffusion on DNA follow a rotation-coupled translation mechanism?

**DOI:** 10.1038/s41467-020-18855-1

**Published:** 2020-10-05

**Authors:** Harry Mark Greenblatt, Haim Rozenberg, Dina Daitchman, Yaakov Levy

**Affiliations:** Department of Structural Biology, Weizmann Institute of Science, 76100 Rehovot, Israel

**Keywords:** Biophysics, Structural biology

**Arising from** De March et al. *Nature Communications* 10.1038/ncomms13935 (2017)

Various DNA-binding proteins were shown, by both experimental and theoretical approaches, to diffuse in a rotation-coupled translation manner^1–4^, while the mechanistic features of this sliding dynamics may vary for different proteins depending on their molecular properties^1,4–7^. In the work by De March et al.^8^, using three different techniques (X-ray crystallography, nuclear magnetic resonance (NMR) measurements, and molecular dynamics (MD) simulations), the authors claim that the toroidal PCNA protein also diffuses along DNA in a motion that is coupled to rotation along the DNA axis. We found that the NMR and MD reported by De March et al. to be inconclusive, and, most importantly, the diffraction data do not support the reported location of the DNA. In light of this, the proposed spiral cog-wheel mechanism, where the clamp keeps a fixed orientation relative to the DNA, as the main component for PCNA diffusion along DNA, is not supported by their work.

De March et al. present a crystal structure of a complex between the homotrimer of human PCNA and a 10 bp stretch of double stranded DNA (originally deposited as PDB 5L7C)^8^. In their model, they identify five interactions between arginine or lysine side chains and the DNA phosphate backbone. These unique interactions are used by the authors to propose that “the orientation of the clamp is invariant relative to DNA” and the PCNA–DNA interface thus supports “a helical sliding mechanism in which the clamp rotates and tilts by keeping a fixed orientation relative to the DNA backbone”^8^. Examining the deposited structure and data, however, we felt that the purported interactions between the protein and the DNA were not supported by the diffraction data, due to extremely high atomic thermal displacement parameters (B-factors) for the DNA duplex, and poor density on the DNA. During our correspondence with the authors while this communication was in preparation, they revised the PDB file (6GIS), indicating that the original submitted version of 5L7C was in error. Our remarks, therefore, are based on this revised coordinate file. In the modified PDB file the average B-factor for the DNA is indeed lower, yet is still ~3.4 times higher relative to that of the protein. Furthermore, the average B-factors for the side-chains of the protein residues that the authors show interacting with the DNA are not lower than their counterparts in other chains. If specific residues of a given subunit are interacting with a ligand, one expects their B-factors to be lower than the equivalent free residues in other subunits due to these stabilizing interactions. This, however, is not the case: the side-chain B-factors of Lys20, Lys217, Lys80, and His153 are similar in all three subunits. In fact, Lys77 and Arg149 of chain A appear even more disordered than their counterparts in other chains. It should also be noted that Arg149 of Chain A does not even form a salt bridge with the DNA, since its polar end is facing away from the DNA backbone. The closest polar atom of the side chain of Arg149 is Nε, at 4.2 Å from the closest phosphate oxygen, too far for a standard H-bond.

Finally, and most importantly, we could not see any difference density (mFo-DFc) in an omit map that could unambiguously locate the DNA in the position reported by the authors. Examining the difference maps (even at 1.3*σ*, as used by De March et al.), we see no density characteristic of B-DNA, such as base-pair stacking or phosphate backbone (Fig. 1a–c). In fact, at 1.3*σ*, there is so much noise, one must ask why the authors decided to place the DNA where they did, while ignoring the rest of the copious density. It may be argued that standard solvent modeling would eliminate the weak density for some low occupancy DNA. To investigate this possibility, we used Phenix to generate a Polder omit map^9^. In Fig. 1c, we show the results of the Polder map; even if one could argue that there is some structural signal, it is at low occupancy and not recognizable as DNA.

**Fig. 1:**
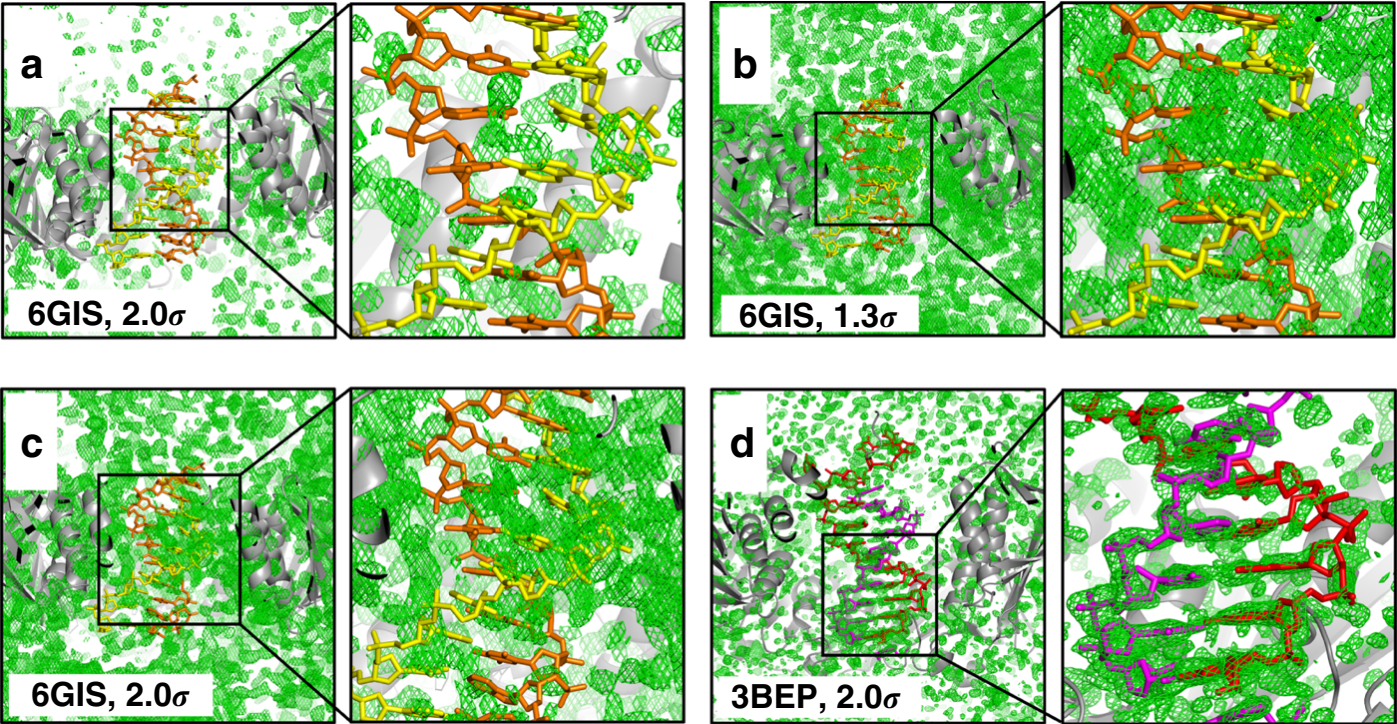
Assessment of the electron density of putative DNA in PDB 6GIS. **a** Simulated annealing difference omit map (mFo-DFc), contoured at 2.0*σ*, overlaid with the model of DNA from the crystal structure, showing no evidence of DNA bound to the interior of PCNA. The DNA strand that interacts with the protein is colored yellow, and the complementary strand is colored orange. **b** Same difference map at 1.3*σ*. Note that the noisy density also covers parts of the protein, indicating that the contour level is too low, but still does not correspond to the DNA. **c** Polder omit map contoured at 1.3*σ*, showing copious density, but not corresponding to DNA, particularly on the lower left of the yellow strand, which purportedly interacts with PCNA. In order to generate an effective omit map, we removed the DNA (7.2% of the total atoms), and ran simulated annealing using PHENIX^11^ to decrease bias from the starting model. We also removed all TLS and NCS parameters, and used group B factors (2 values per residue, in consideration of the relatively low resolution: 2.82 Å), but no other adjustments were made to the model. This resulted in a significant drop in both Rwork (from 24.7 to 19.5%) and Rfree (from 28.4 to 24.9%), and the final geometry was also improved (RMSD bonds from 0.016 to 0.008Å, and RMSD angles from 1.8 to 1.0°), clearly indicating a large improvement in the model. The RMSD between the starting structure and the final structure was 0.51 Å for all protein atoms. In order to confirm that the improvement in Rwork and Rfree were not resulting from differences between Phenix and REFMAC^12^, the output from Phenix was run through REFMAC, giving a final R-work of 19.1%, and Rfree of 24.6%. **d** Simulated annealing difference omit map, for 3BEP, contoured at 2*σ*. Images made in PyMOL.

As a control, we performed a similar analysis using the *E. coli* sliding clamp on DNA (3BEP)^10^ by removing DNA from the structure and running simulated annealing. In the resultant difference map (Fig. 1d), despite the poor quality of the density, at 2*σ* the distinctive shape of stacked bases is clear, indicating the presence of DNA. This is in contrast to the results for 6GIS.

Given these observations, placement of duplex DNA at its published location, especially at 100% occupancy, is therefore not justified by the X-ray crystallographic analysis of De March et al.^8^ As such, their claims about specific residues interacting with the DNA have no structural basis. De March et al. comment on “the existence of a subpopulation of complexes with slightly different DNA orientations” to explain the high thermal parameters and the potential exchange between symmetric sites of PCNA. This uncertainty, however, in the DNA orientation is highly underestimated, and should at least be reflected in significantly lower occupancy for the DNA. Furthermore, exchange between symmetrical sites on the three PCNA subunits can hardly be termed “slightly different” orientations.

The NMR data show interactions between the residues that line the inner ring of PCNA and the DNA, but these observations are consistent with interactions that are distributed symmetrically around the inner ring. These interactions could be formed simultaneously by centrally located DNA, by transient interactions as the DNA moves around the inner channel, or a combination of both. This would allow for simple linear translation of the DNA, without requiring rotation. All these mechanisms are governed by transient PCNA–DNA interactions which are consistent with the very high *K*_D_ (~0.7 mM).

The MD simulations based on the 5L7C hPCNA–DNA complex suggests that the five interfacial salt-bridges are formed throughout the 250 ns simulation, but this might be a direct consequence of using the biased experimental model as the initial structure for the simulations. No direct diffusion, however, is shown in this simulation, most probably because of the short timescale, rendering their results inconclusive^8^.

Coarse-grained molecular dynamics simulations and electrostatic free energy calculations support a weak PCNA–DNA interface and therefore one-dimensional diffusion of PCNA along DNA should occur via translocation that is uncoupled from rotation where the DNA is placed, on average, at the center of the PCNA inner ring^13^. Obviously, direct interactions between PCNA and DNA are, in principle, possible but they are expected to be transient and not lead to sustained helical motion of PCNA along DNA. Further support for uncoupled diffusion of PCNA on DNA comes from a single-molecule experiment on the dependence of the diffusion coefficient of PCNA on its dimension^14^, which is very similar to that of the TALE protein that was concluded to follow uncoupled rotation-translation diffusion along DNA^15^. The former single-molecule study, however, reported that upon changing the solvent viscosity PCNA may track the DNA helical pitch^14^. These contradictory results, which were seemingly resolved following simple modeling by favoring the coupled rotation–translation diffusion of PCNA along DNA, demand further investigation of PCNA, as well as other toroidal proteins, to resolve the unique properties of their interactions with DNA and their ambiguous characteristics.

In conclusion, the proposed mechanism of translation-coupled rotation as the main mode of PCNA sliding along the DNA backbone, implies sustained contacts with the DNA by five residues of one single subunit, with stochastic exchange among adjacent phosphate atoms. This mechanism is at odds with the lack of electron density for DNA in the crystal structure, and the observed low binding affinity of PCNA for DNA. Furthermore, the lack of rotation in the MD simulations, and the inconclusive NMR data do not support this mechanism. While using complementary approaches to provide a fuller and consistent picture is commendable, we do not find that any of the results presented by De March et al. support the contention that PCNA slides in a rotation-coupled fashion.

## Data Availability

No datasets were generated for this manuscript.
